# Supplementary material for: Metabolomic Analysis of Trehalose Alleviating Oxidative Stress in Myoblasts
Source: Int J Mol Sci. 2023 Aug 28;24(17):13346. doi: 10.3390/ijms241713346 (PMC10488301; doi:10.3390/ijms241713346)
Supplement: Supplementary file 1 [file ijms-24-13346-s001.zip › ijms-2563904-supplementary.pdf]

Supplementary Materials

Figure S1

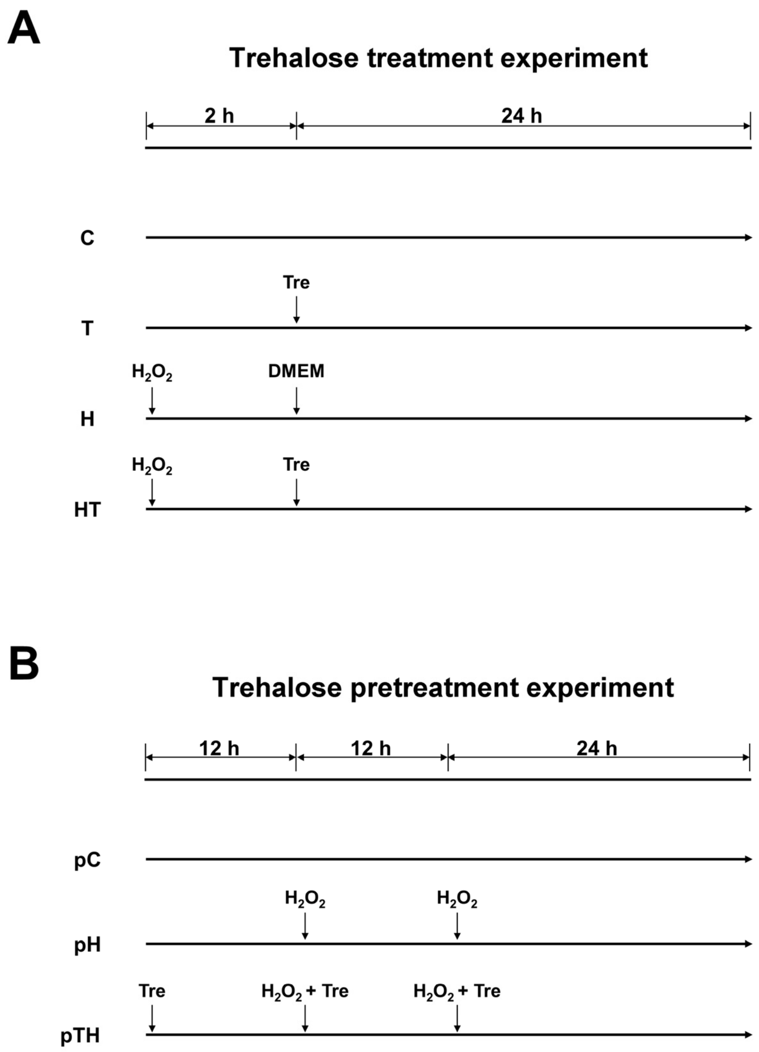

Figure S1. Schematic diagrams of cell experimental design. H_2O_2 and trehalose were used at final concentrations of 200 μM and 10 mM, respectively. **(A)** The trehalose treatment experiment for addressing the therapeutic effect of trehalose on C2C12 cells under short-term oxidative stress. Four groups (H, HT, C, T) of cells were used: the H cells were cultured in DMEM supplemented with H_2O_2 for 2 h, then continuously cultured in DMEM for 24 h; the HT cells were cultured in DMEM supplemented with H_2O_2 for 2 h, then continuously cultured in DMEM supplemented with trehalose for 24 h; the C and T cells were cultured in DMEM or DMEM supplemented with trehalose, respectively without exposure to H_2O_2 for 24 h. Abbreviations: C, normal control; T, trehalose

treatment; H, short-term H_2O_2 exposure; HT, short-term H_2O_2 exposure + trehalose treatment. **(B)** The trehalose pretreatment experiment for addressing the preventive effect of trehalose on C2C12 cells under prolonged oxidative stress. Three groups (pTH, pH, pC) of cells were used: the pTH cells were cultured in DEME supplemented with trehalose for 12 h, then continuously cultured in DEME supplemented with H_2O_2 and trehalose for 12 h, followed by an additional 24-h culture; the pH cells were cultured in DEME for 12 h, then continuously cultured in DEME supplemented with H_2O_2 for 12 h, followed by an additional 24 h culture; the pC cells were cultured in DEME for 12 h, then continuously cultured in DEME for 12 h, followed by an additional 24 h culture without exposure to H_2O_2. Abbreviations: pC, normal control; pH, prolonged H_2O_2 exposure; pTH, prolonged H_2O_2 exposure + trehalose pretreatment.

Figure S2

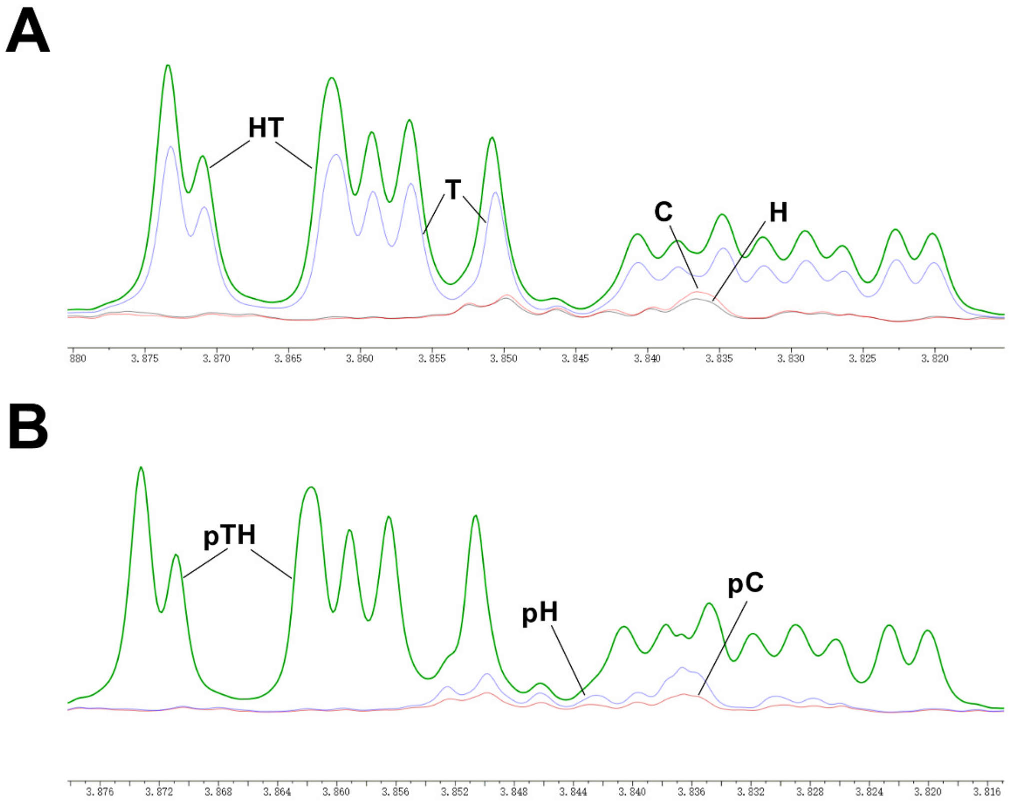

Figure S2. Local amplified regions of trehalose peaks in typical 1D ^{1}H-NMR spectra recorded on aqueous metabolites from C2C12 cells. (A) Part of trehalose peaks (δ 3.81-3.88) in the C, T, H and HTcells. Red line: spectral region from the C group. Lilac line: spectral region from the T group. Grey line: spectral region from the H cells. Green line: spectral region from the HT cells**. (B)** Part of trehalose peaks (δ 3.81-3.88) in the pC, pH and pTH cells. Red line: spectral region from the pC cells. Lilac line: spectral region from the pH cells. Green line: spectral region from the pTH cells**.** The TSP peak was used as a chemical shift reference (δ 0.00).

Figure S3

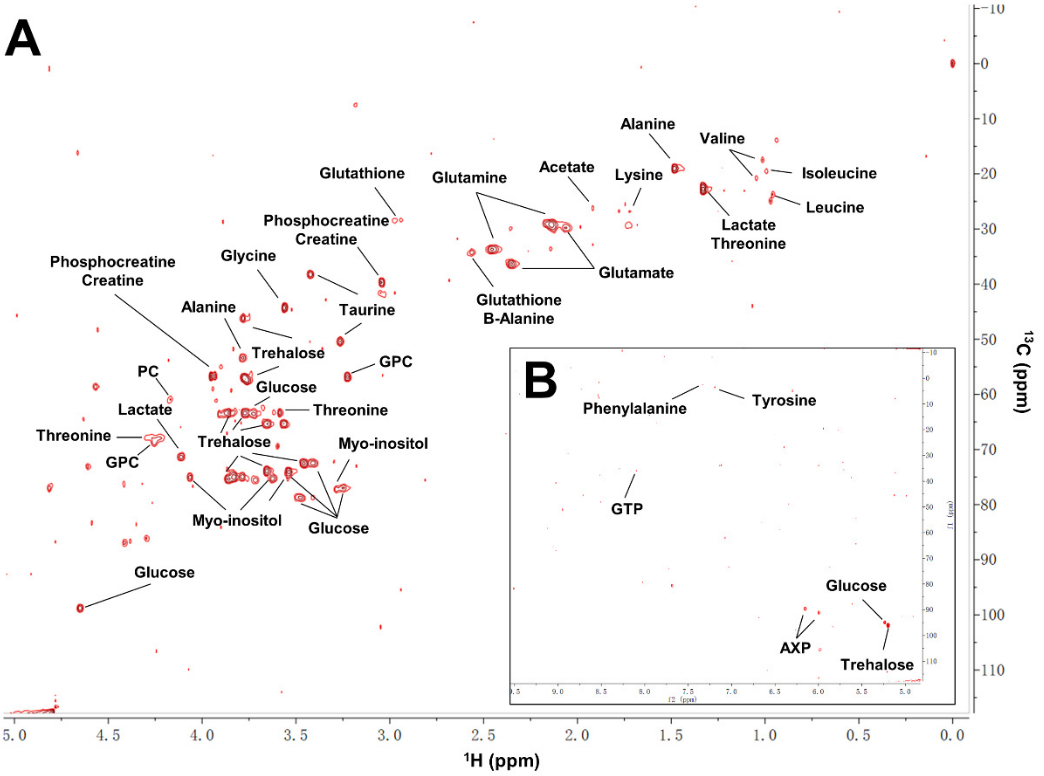

Figure S3. Representative 2D ^{1}H-^{13}C HSQC spectra of aqueous metabolites from C2C12 cells treated with trehalose under short-term oxidative stress (the HT cells). The spectral ranges of **(A)** 0.8-5.0 ppm and **(B)** 5.0-9.5 ppm are shown separately.

Figure S4

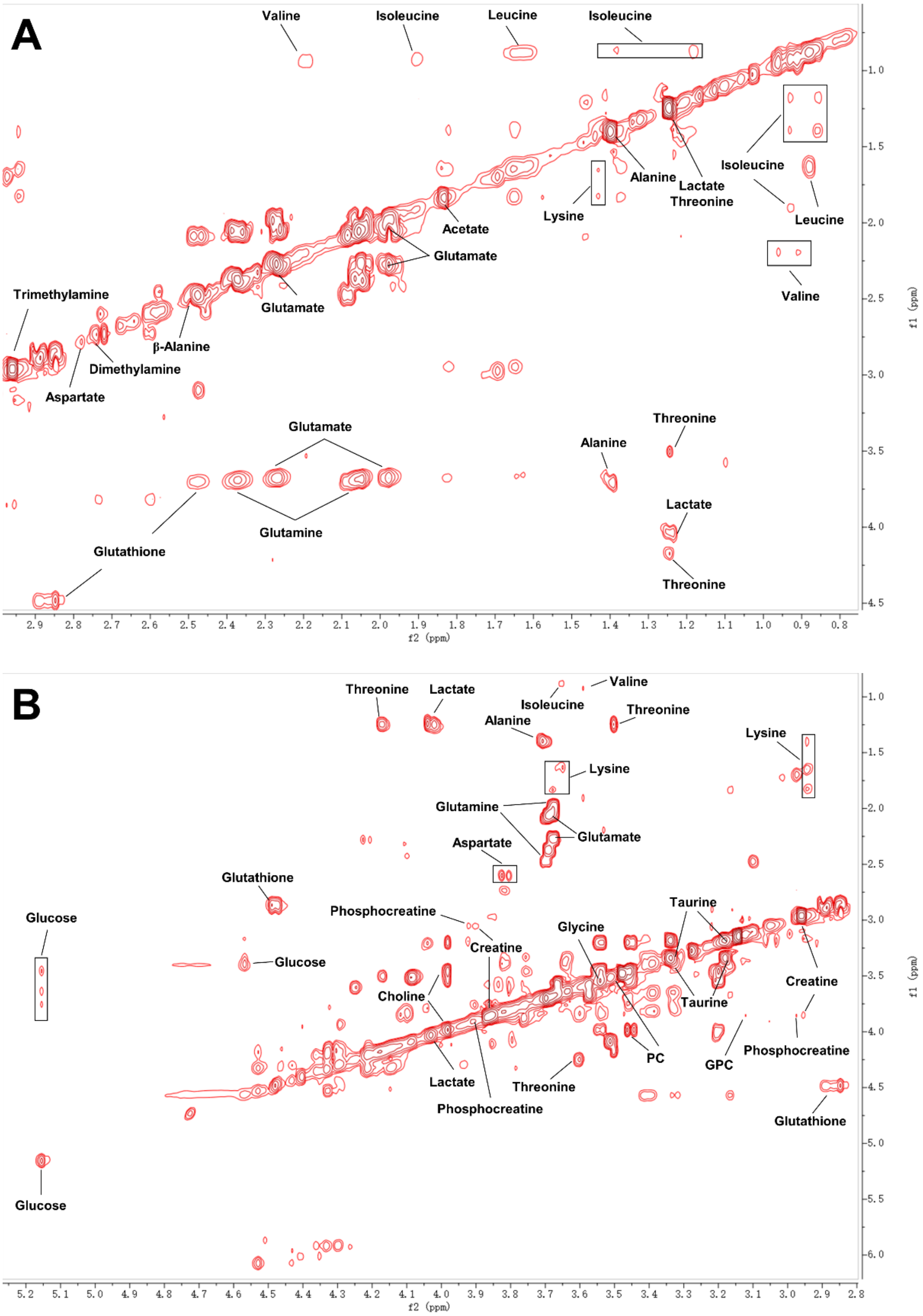
A
Valine
Isoleucine
Leucine
Isoleucine
Isoleucine
Leucine
Valine
Alanine
Lactate
Threonine
Lysine
Acetate
Glutamate
Glutamate
Trimethylamine
β-Alanine
Dimethylamine
Aspartate
Glutamate
Threonine
Alanine
Lactate
Threonine
Glutamine
Glutathione
f2 (ppm)
f1 (ppm)
B
Threonine
Lactate
Valine
Isoleucine
Threonine
Alanine
Lysine
Lysine
Glutamine
Glutamate
Aspartate
Glutathione
Glucose
Phosphocreatine
Taurine
Glycine
Creatine
Glucose
Choline
Taurine
Creatine
PC
GPC
Phosphocreatine
Lactate
Threonine
Phosphocreatine
Glutathione
Glucose
f2 (ppm)
f1 (ppm)

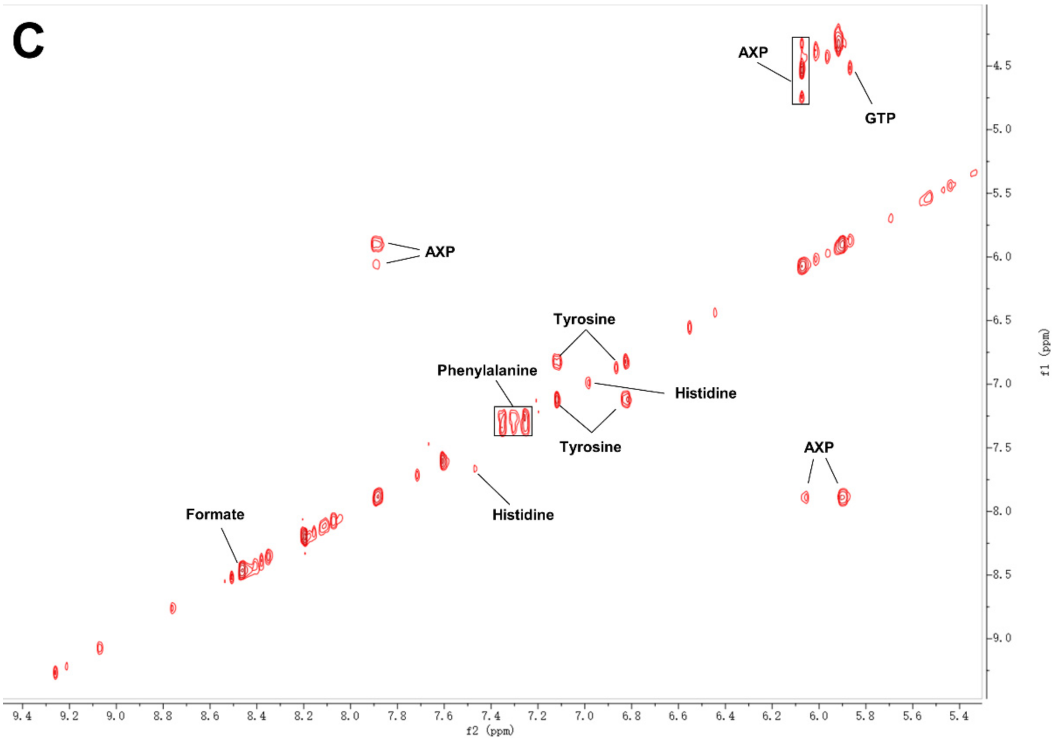

Figure S4. Representative 2D ^{1}H-^{1}H TOCSY spectra of aqueous metabolites from C2C12 cells treated with trehalose under short-term oxidative stress (the HT cells). The spectral ranges of **(A)** 0.8-3.0 ppm, **(B)** 3.0-5.3 ppm and **(C)** 5.3-9.4 ppm are shown separately.

Figure S5

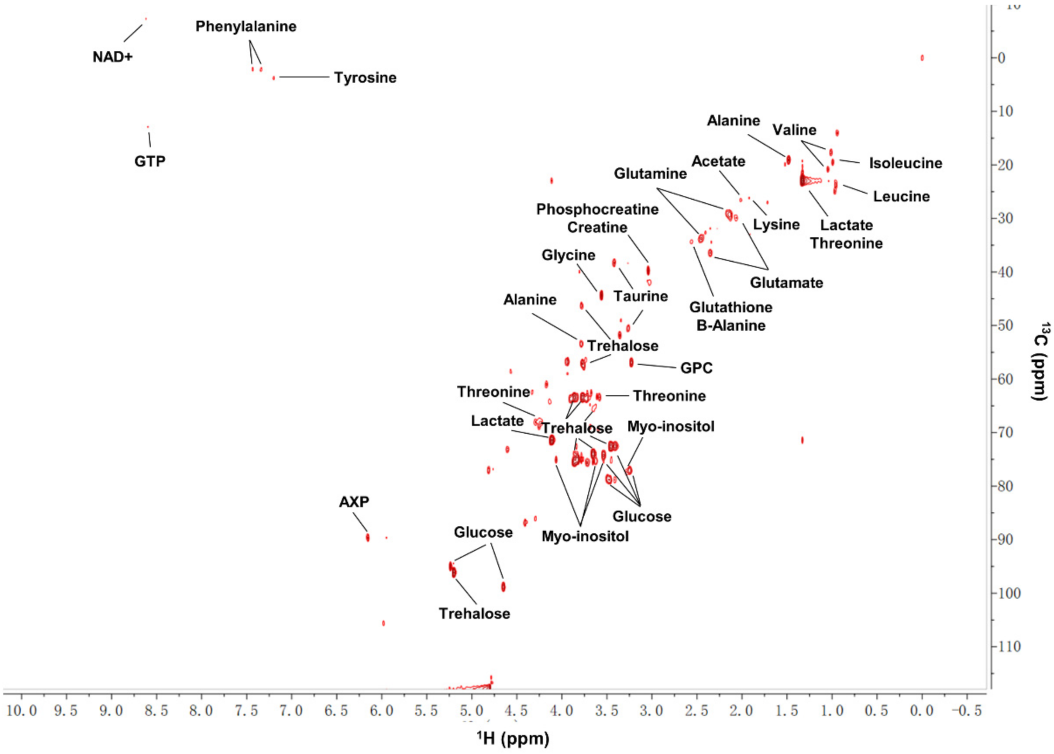

Figure S5. Representative 2D ^{1}H-^{13}C HSQC spectrum of aqueous metabolites from C2C12 cells pretreated with trehalose under prolonged oxidative stress (the pTH cells).

Figure S6

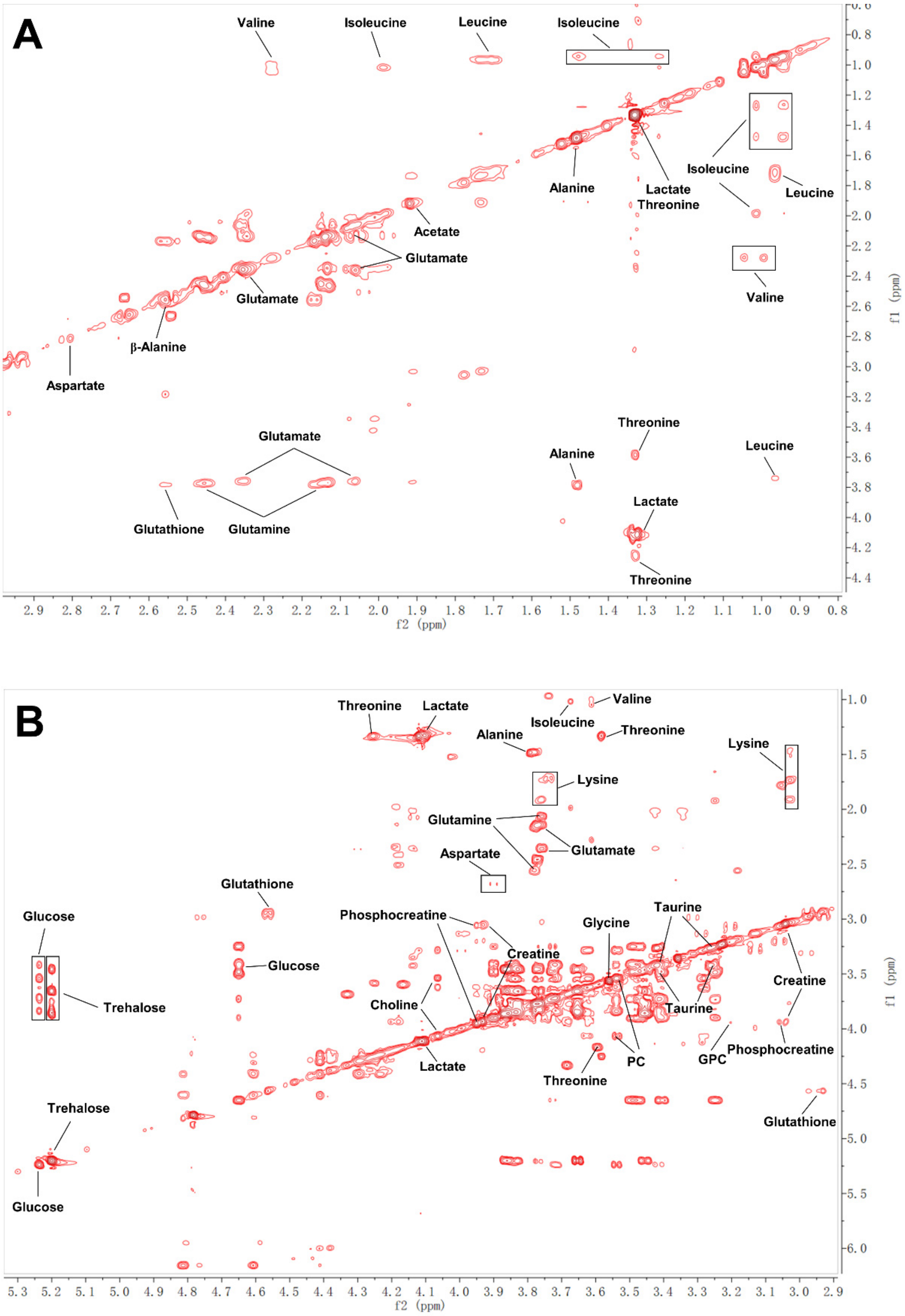
A
Valine
Isoleucine
Leucine
Isoleucine
Isoleucine
Leucine
Alanine
Lactate
Threonine
Acetate
Glutamate
Glutamate
Valine
β-Alanine
Aspartate
Threonine
Leucine
Glutamate
Alanine
Lactate
Glutathione
Glutamine
Threonine
f2 (ppm)
f1 (ppm)
B
Threonine
Lactate
Valine
Isoleucine
Alanine
Threonine
Lysine
Lysine
Glutamine
Glutamate
Aspartate
Glutathione
Glucose
Phosphocreatine
Glycine
Taurine
Creatine
Glucose
Trehalose
Choline
Creatine
Taurine
Lactate
PC
GPC
Phosphocreatine
Threonine
Trehalose
Glutathione
Glucose
f2 (ppm)
f1 (ppm)

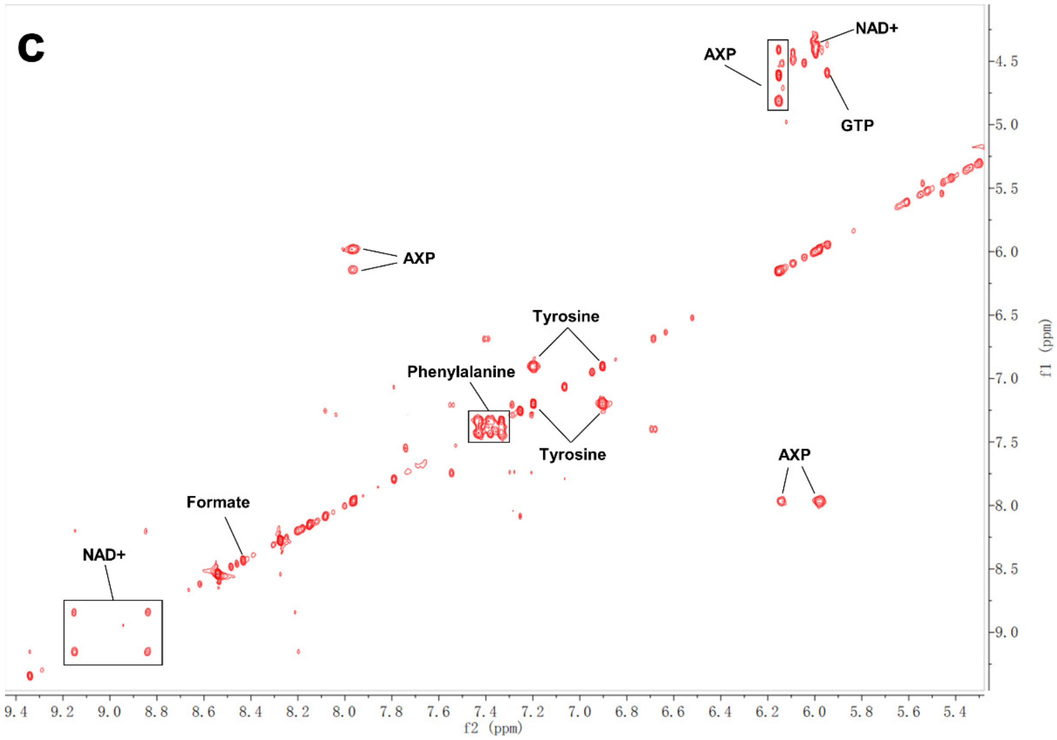

Figure S6. Representative 2D ^{1}H-^{1}H TOCSY spectra of aqueous metabolites from C2C12 cells pretreated with trehalose under prolonged oxidative stress (the pTH cells). The spectral ranges of **(A)** 0.8-3.0 ppm, **(B)** 3.0-5.3 ppm and **(C)** 5.3-9.4 ppm are shown separately.

Figure S7

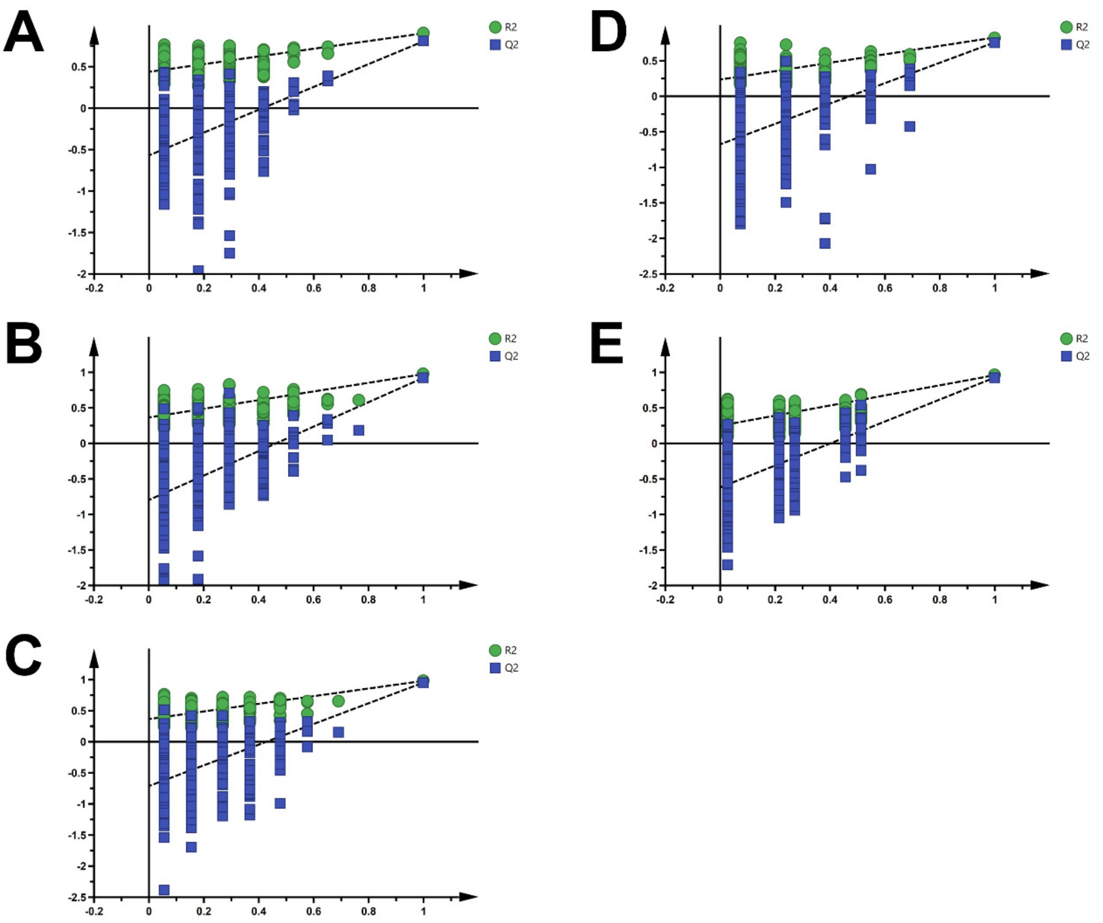

Figure S7. Cross-validation plots of the OPLS-DA models for the 1D ^{1}H-NMR spectral data of C2C12 cells. (A) H *vs.* C (R2Y = 0.903, Q2Y = 0.808), **(B)** HT *vs.* H (R2Y = 0.974, Q2Y = 0.923), **(C)** T *vs.* C (R2Y = 0.980, Q2Y = 0.953), **(D)** pH *vs.* pC (R2Y = 0.828, Q2Y = 0.759), and **(E)** pTH *vs.* pH (R2Y = 0.960, Q2Y = 0.927). The cross-validation plots were obtained by response permutation tests using the first two components (n = 200).

Figure S8

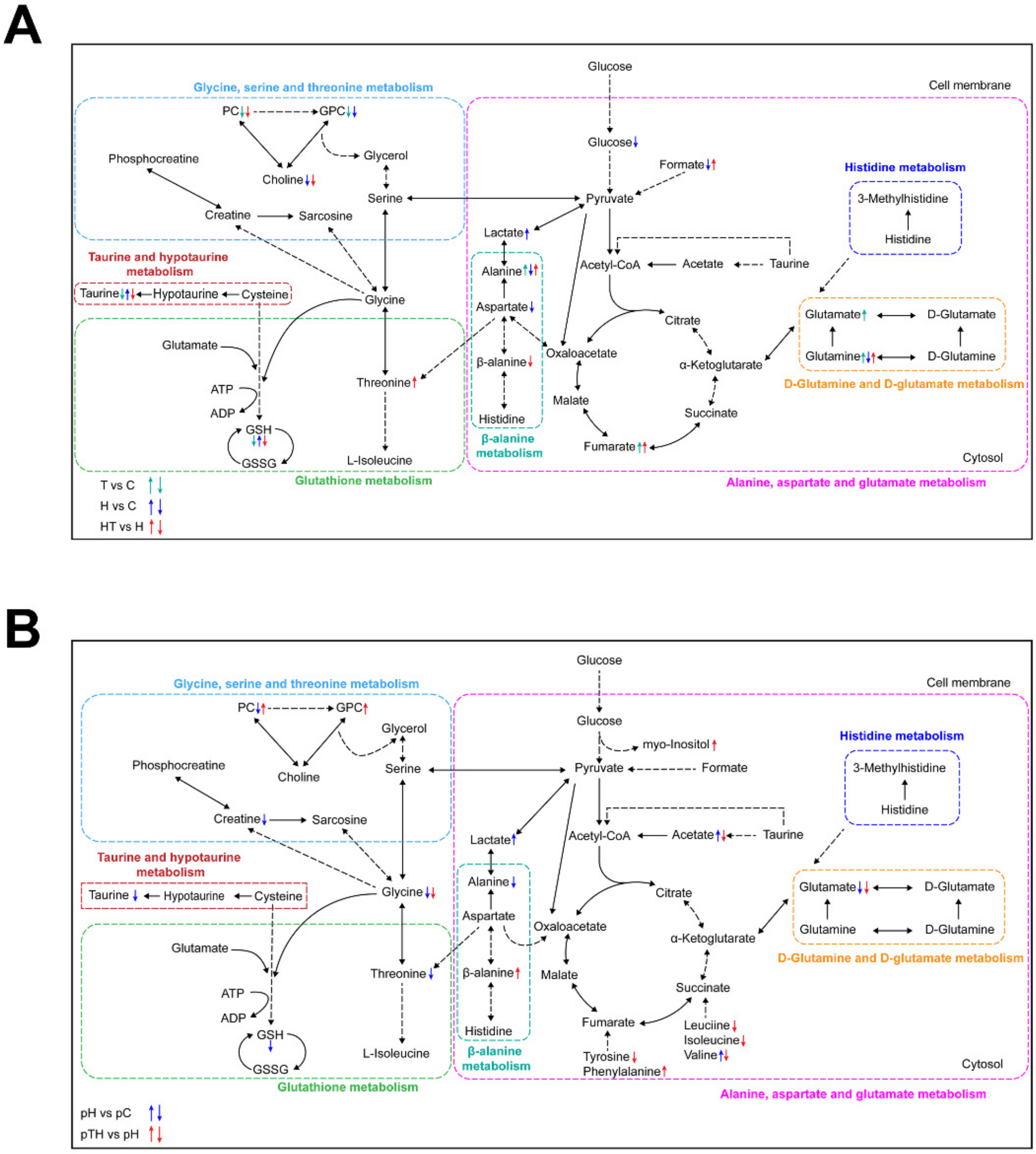

Figure S8. Schematic representation of the changing trends of metabolites in the significantly altered metabolic pathways of C2C12 cells under the intervention of H_2O_2 and trehalose. (A) Trehalose treatment shows a therapeutic effect against short-term oxidative stress; (**B**) Trehalose pretreatment shows a preventive effect against prolonged oxidative stress. Dotted arrows represent multi-step reactions and solid arrows represent one-step reactions. The up/down arrow highlights the increased/decreased metabolites. Abbreviations: PC, O-phosphocholine; GPC, sn-glycerol-3-phosphate choline; ATP, adenine nucleoside triphosphate; ADP, adenosine diphosphate; GSH, reduced glutathione; GSSG, oxidized glutathione.

Table S1. Identification of aqueous metabolites derived from the C, T, H and HT groups of C2C12 cells.

Metabolite	δ ^{1}H(ppm) and multiplicity	Moieties
Pantothenate	0.88(s), 0.92(s)	CH_3, CH_3
Leucine	0.96(d), 0.97(d), 1.69(m), 1.70(m), 1.73(m), 3.73(m)	α-CH_3, α-CH_3, γ-CH, α-CH_3, β-CH_2, α-CH
Isoleucine	0.94(t), 1.01(d), 1.21(m), 1.42(m), 2.00(m), 3.67(d)	δ-CH_3, γ-CH_3, half γ-CH_2, half γ-CH_2, β-CH, α-CH
Valine	0.99(d), 1.05(d), 2.26(m), 3.60(d)	γ-CH_3, γ-CH_3, β-CH, α-CH
Fucose	1.22(d), 1.26(d), 3.45(dd), 3.65(dd), 3.78(m), 3.86(dd), 4.20(q), 4.56(d), 5.21(d)	CH, CH_3, CH-O, O-CH-O
Alanine	1.47(d), 3.78(q)	β-CH_3, α-CH
Acetate	1.91(s)	CH_3
Proline	1.99(m)	γ-CH_2
Glutamate	2.08(m), 2.12(m), 2.34(m), 2.37(m), 3.75(m)	half β-CH_2, half β-CH_2, half γ-CH_2, half γ-CH_2, α-CH
Pyroglutamate	2.05(m), 2.39 (d), 2.51 (m), 4.18(dd)	β-CH, γ-CH_2, β-CH, α-CH
Glutamine	2.13(m), 2.45(m), 3.77(t)	γ-CH_2, β-CH_2, α-CH
Dimethylamine	2.50(s)	CH_3
Trimethylamine	2.88(s)	CH_3
Aspartate	2.68(dd), 2.81(dd), 3.90(dd)	β-CH_2, α-CH
Glutathione	2.15(m), 2.55(m), 2.96(m), 3.77(m), 4.56(m)	β-CH_2, γ-CH_2, CH_2-SH, α-CH&CH_2-NH, CH-NH
Lysine	1.89(m), 1.92(m), 3.02(t), 3.75(t)	γ-CH2, half γ-CH2, δ-CH2, β-CH2, ε-CH2, α-CH
Creatine	3.04(s), 3.93(s)	N-CH_3, α-CH_2
Creatinephosphate	3.05(s), 3.94(s)	N-CH_3, CH_2

Beta-Alanine	2.54(t), 3.17(t)	CH_2, CH_2
Choline	3.20(s), 3.50(dd), 4.03(t)	N-$(CH_3)_3$, N-CH_2, CH_2OH
PC	3.22(s), 3.60(t), 4.18(m)	N-$(CH_3)_3$, N-CH_2 , CH_2OH
GPC	3.23 (s), 3.60 (dd), 3.68 (dd), 3.87 (m), 3.94 (m), 4.33 (m)	N–$(CH_3)_3$, half $^1CH_2$, $^2CH_2$, half $^2CH_2$, half $^3CH_2$, half $^3CH_2$, $^1CH_2$
Taurine	3.24(t),3.41(t)	$^1CH_2$, $^2CH_2$
Glucose	β(3.24(dd), 3.48(t), 3.90(dd)), α(3.54(dd), 3.71(t), 3.72(dd), 3.83(m))	β(H_2, H_3, H_5), α(H_2, H_3, H_6)
Glycine	3.57(s)	α-CH_2
Threonine	1.33(d), 3.59(d), 4.25(m)	γ-CH_2, β-CH
myo-Inositol	3.28(t), 3.53(dd), 3.63(t), 4.07(t)	^2CH, $^{4,6}CH$, $^{1,3}CH$, ^5CH
Lactate	1.33(d), 4.11(q)	β-CH_3, α-CH
Fumarate	6.51(s)	CH
Histidine	7.06(s), 7.85(s)	^5CH, ^2CH
Tyrosine	3.05(dd), 3.19(dd), 6.92(d), 7.19(d)	half β-CH_2, half β-CH_2, β-CH, α-CH
Phenylalanine	3.12(dd), 3.30(dd), 3.99(dd), 7.33(d), 7.37(t), 7.43(t)	α-CH, half β-CH_2, half β-CH_2, α-CH, β-CH, γ-CH
GTP	5.92 (d), 8.1 (s)	CH, CH
AXP	6.14(d), 8.27(s), 8.58(s)	NH_2 , δ-CH, ^2CH
Formate	8.46(s)	CH
Trehalose	3.42(dd), 3.49(dd), 3.64(dd), 3.75(dd), 3.81(dt), 3.75(m), 3.85(dt), 3.85(dd), 3.86(dd), 4.12(dd), 5.18(dt)	δ-CH, β-CH, ^5CH, α-CH

s, singlet; d, doublet; t, triplet; q, quartet; m, multiplet; dd, doublet of doublets; dt, doublet of triplets.

(PC, O-phosphate choline; GPC, sn-glycerol-3-phosphate choline; GTP, guanosine triphosphate; AXP, adenine mono/di/triphosphate)

Table S2. Identification of aqueous metabolites from the pC, pH and pTH groups of C2C12 cells.

Metabolite	δ ^{1}H(ppm) and multiplicity	Moieties
Pantothenate	0.88(s), 0.92(s)	CH_3, CH_3
Leucine	0.96(d), 0.97(d), 1.69(m), 1.70(m), 1.73(m), 3.73(m)	α-CH_3, α-CH_3, γ-CH, α-CH_3, β-CH_2, α-CH
Isoleucine	0.94(t), 1.01(d), 1.21(m), 1.42(m), 2.00(m), 3.67(d)	δ-CH_3, γ-CH_3, half γ-CH_2, half γ-CH_2, β-CH, α-CH
Valine	0.99(d), 1.05(d), 2.26(m), 3.60(d)	γ-CH_3, γ-CH_3, β-CH, α-CH
Ethanol	1.17(t), 3.65(q)	δ-CH_3, CH_2
Fucose	1.22(d), 1.26(d), 3.45(dd), 3.65(dd), 3.78(m), 3.86(dd), 4.20(q), 4.56(d), 5.21(d)	CH, CH_3, CH-O, O-CH-O
Alanine	1.47(d), 3.78(q)	β-CH_3, α-CH
Acetate	1.91(s)	CH_3
Proline	1.99(m)	γ-CH_2
Glutamate	2.08(m), 2.12(m), 2.34(m), 2.37(m), 3.75(m)	half β-CH_2, half β-CH_2, half γ-CH_2, half γ-CH_2, α-CH
Pyroglutamate	2.05(m), 2.39 (d), 2.51 (m), 4.18(dd)	β-CH, γ-CH_2, β-CH, α-CH
Glutamine	2.13(m), 2.45(m), 3.77(t)	γ-CH_2, β-CH_2, α-CH
Dimethylamine	2.50(s)	CH_3
Trimethylamine	2.88(s)	CH_3
Aspartate	2.68(dd), 2.81(dd), 3.90(dd)	β-CH_2, α-CH
Glutathione	2.15(m), 2.55(m), 2.96(m), 3.77(m), 4.56(m)	β-CH_2, γ-CH_2, CH_2-SH, α-CH&CH_2-NH, CH-NH
Lysine	1.89(m), 1.92(m), 3.02(t), 3.75(t)	γ-CH2, half γ-CH2, δ-CH2, β-CH2, ε-CH2, α-CH
Creatine	3.04(s), 3.93(s)	N-CH_3, α-CH_2

Creatinephosphate	3.05(s), 3.94(s)	N-CH_3, CH_2
Beta-Alanine	2.54(t), 3.17(t)	CH_2, CH_2
Choline	3.20(s), 3.50(dd), 4.03(t)	N-$(CH_3)_3$, N-CH_2, CH_2OH
PC	3.22(s), 3.60(t), 4.18(m)	N-$(CH_3)_3$, N-CH_2 , CH_2OH
GPC	3.23 (s), 3.60 (dd), 3.68 (dd), 3.87 (m), 3.94 (m), 4.33 (m)	N–$(CH_3)_3$, half $^1CH_2$, $^2CH_2$, half $^2CH_2$, half $^3CH_2$, half $^3CH_2$, $^1CH_2$
Taurine	3.24(t),3.41(t)	$^1CH_2$, $^2CH_2$
Glucose	β(3.24(dd), 3.48(t), 3.90(dd)), α(3.54(dd), 3.71(t), 3.72(dd), 3.83(m))	β(H_2, H_3, H_5), α(H_2, H_3, H_6)
Glycine	3.57(s)	α-CH_2
Threonine	1.33(d), 3.59(d), 4.25(m)	γ-CH_2, β-CH
myo-Inositol	3.28(t), 3.53(dd), 3.63(t), 4.07(t)	^2CH, $^{4,6}CH$, $^{1,3}CH$, ^5CH
Lactate	1.33(d), 4.11(q)	β-CH_3, α-CH
Fumarate	6.51(s)	CH
Histidine	7.06(s), 7.85(s)	^5CH, ^2CH
Tyrosine	3.05(dd), 3.19(dd), 6.92(d), 7.19(d)	half β-CH_2, half β-CH_2, β-CH, α-CH
Phenylalanine	3.12(dd), 3.30(dd), 3.99(dd), 7.33(d), 7.37(t), 7.43(t)	α-CH, half β-CH_2, half β-CH_2, α-CH, β-CH, γ-CH
GTP	5.92 (d), 8.1 (s)	CH, CH
AXP	6.14(d), 8.27(s), 8.58(s)	NH_2 , δ-CH, ^2CH
Formate	8.46(s)	CH
Trehalose	3.42(dd), 3.49(dd), 3.64(dd), 3.75(dd), 3.81(dt), 3.75(m), 3.85(dt), 3.85(dd), 3.86(dd), 4.12(dd), 5.18(dt)	δ-CH, β-CH, ^5CH, α-CH

s, singlet; d, doublet; t, triplet; q, quartet; m, multiplet; dd, doublet of doublets; dt, doublet of triplets. (PC, O-phosphate choline; GPC, sn-glycerol-3-phosphate choline; GTP, guanosine triphosphate; AXP, adenine mono/di/triphosphate)

Table S3. Significant metabolites identified from the pairwise comparisons of the C, T, H and HT groups of C2C12 cells.

**No.**	**metabolite**	**H *vs.* C**	**T *vs.* C**	**HT *vs.* H**
1	Taurine	√	√	√
2	Glutamine	√	√	√
3	Formate	√	√	√
4	Glutathione	√	√	
5	Aspartate	√	√	
6	GPC	√	√	
7	AXP	√	√	
8	Proline	√	√	
9	Choline	√		√
10	Dimethylamine	√		√
11	Fucose	√		√
12	Alanine		√	√
13	Pyroglutamate		√	√
14	PC		√	√
15	Lysine		√	√
16	Fumarate		√	√
17	Trimethylamine	√		
18	Lactate	√		
19	Glucose	√		
20	Histidine	√		
21	Glutamate		√	
22	Pantothenate		√	
23	Beta-Alanine			√
24	Threonine			√

Table S4. Significant metabolites identified from the pairwise comparisons of the pC, pH and pTH groups of C2C12 cells.

No.	metabolite	pH *vs.* pC	pTH *vs.* pH
1	Glutamate	√	√
2	Valine	√	√
3	Creatine	√	√
4	Tyrosine	√	√
5	Leucine	√	√
6	Isoleucine	√	√
7	PC	√	√
8	Acetate	√	
9	Lactate	√	
10	AXP	√	
11	GTP	√	
12	Glutathione	√	
13	Taurine	√	
14	Lysine	√	
15	Glucose	√	
16	Phenylalanine	√	
17	Alanine	√	
18	myo-Inositol		√
19	Beta-Alanine		√
20	Tryptophan		√
21	Glycine		√
22	Proline		√

Table S5. Differential metabolites identified from the pairwise comparisons of the C, T, H and HT groups of C2C12 cells.

No.	metabolites	H *vs.* C	T *vs.* C	HT *vs.* H
1	Alanine	√	√	√
2	Glutamine	√	√	√
3	Glutathione	√	√	√
4	Taurine	√	√	√
5	GPC	√	√	
6	Choline	√		√
7	Formate	√		√
8	Pyroglutamate		√	√
9	PC		√	√
10	Fumarate		√	√
11	Proline		√	
12	Aspartate	√		
13	Dimethylamine	√		
14	Trimethylamine	√		
15	Glucose	√		
16	AXP	√		
17	Lactate	√		
18	Lysine		√	
19	Pantothenate		√	
20	Glutamate		√	
21	Beta-Alanine			√
22	Threonine			√

Table S6. Differential metabolites identified from the pairwise comparisons of the pC, pH and pTH groups of C2C12 cells.

No.	metabolite	pH *vs.* pC	pTH *vs.* pH
1	Acetate	√	√
2	Glutamate	√	√
3	Glycine	√	√
4	PC	√	√
5	Valine	√	√
6	Lactate	√	
7	Alanine	√	
8	Glutathione	√	
9	Lysine	√	
10	Creatine	√	
11	Taurine	√	
12	Threonine	√	
13	GTP	√	
14	AXP	√	
15	NAD+	√	
16	myo-Inositol		√
17	Leucine		√
18	Isoleucine		√
19	Beta-Alanine		√
20	GPC		√
21	Phenylalanine		√
22	Tryptophan		√
23	Tyrosine		√

Table S7. Characteristic metabolites identified from the pairwise comparisons of the C, T, H and HT groups of C2C12 cells.

No.	metabolite	H *vs.* C	HT *vs.* H	T *vs.* C
1	Glutamine	√	√	√
2	Taurine	√	√	√
3	Glutathione	√		√
4	GPC	√		√
5	Formate	√	√	
6	Alanine		√	√
7	Pyroglutamate		√	√
8	Aspartate	√		
9	Dimethylamine	√		
10	Trimethylamine	√		
11	Glucose	√		
12	AXP	√		
13	Lactate	√		
14	Choline	√		
15	Pantothenate			√
16	Proline			√
17	Glutamate			√
18	Beta-Alanine		√	
19	Threonine		√	

Table S8. Characteristic metabolites identified from the pairwise comparisons of the pC, pH and pTH groups of C2C12 cells.

**No.**	**metabolite**	**pH *vs*. pC**	**pTH *vs*. pH**
1	Glutamate	√	√
2	PC	√	√
3	Valine	√	√
4	Lactate	√	
5	Alanine	√	
6	Glutathione	√	
7	Acetate	√	
8	Lysine	√	
9	Creatine	√	
10	Taurine	√	
11	GTP	√	
12	AXP	√	
13	Glycine		√
14	Myo-inositol		√
15	Leucine		√
16	Isoleucine		√
17	Beta-Alanine		√
18	Tryptophan		√
19	Tyrosine		√

Table S9 Significantly altered metabolic pathways in H_2O_2-exposed C2C12 cells treated with trehalose under short-term oxidative stress.

No.	Significant metabolic pathway	PIV	*p*-value		
			H *vs.* C	HT *vs.* H	T *vs.* C
P1	alanine, aspartate and glutamate metabolism	0.537	2.800×10^{-2}	7.568×10^{-7}	7.201×10^{-9}
P2	D-Glutamine and D-glutamate metabolism	0.500	-	1.761×10^{-5}	1.127×10^{-5}
P3	taurine and hypotaurine metabolism	0.429	4.453×10^{-9}	5.070×10^{-5}	6.961×10^{-6}
P4	beta-Alanine metabolism	0.399	2.000×10^{-2}	4.300×10^{-2}	-
P5	glutathione metabolism	0.371	-	9.403×10^{-5}	3.366×10^{-6}
P6	glycine, serine and threonine metabolism	0.295	3.400×10^{-2}	3.586×10^{-5}	-
P7	histidine metabolism	0.221	1.900×10^{-2}	-	1.216×10^{-3}

[a] Metabolic pathway analysis was performed on the MetaboAnalyst 5.0 webserver, using a combination of metabolite sets enrichment analysis with a criterion of statistical significance $p < 0.05$ and pathway topological analysis with a criterion of pathway impact value (PIV) > 0.2.

[b] These metabolic pathways are all ranked in descending order according to their PIVs.

[c] Blank cells denote $p > 0.05$.

Table S10. Significantly altered metabolic pathways in H_2O_2-exposed C2C12 cells pretreated with trehalose under prolonged oxidative stress.

No.	Significant metabolic pathway	PIV	*p*-value	
			**pH *vs.* pC**	**pTH *vs.* pH**
P8	Phenylalanine, tyrosine and tryptophan biosynthesis	1.000	0.016	0.003
P1	Alanine, aspartate and glutamate metabolism	0.537	0.007	0.023
P2	D-Glutamine and D-glutamate metabolism	0.500	0.011	0.001
P3	Taurine and hypotaurine metabolism	0.429	0.005	-
P4	beta-Alanine metabolism	0.399	-	0.015
P5	Glutathione metabolism	0.371	0.005	0.002
P9	Phenylalanine metabolism	0.357	0.016	0.003
P6	Glycine, serine and threonine metabolism	0.295	0.009	0.001

[a] Metabolic pathway analysis was performed on the MetaboAnalyst 5.0 webserver, using a combination of metabolite sets enrichment analysis with a criterion of statistical significance $p < 0.05$ and pathway topological analysis with a criterion of pathway impact value (PIV) > 0.2.

[b] These metabolic pathways are all ranked in descending order according to their PIVs.

[c] Blank cells denote $p > 0.05$.
